# Automatic schizophrenic discrimination on fNIRS by using complex brain network analysis and SVM

**DOI:** 10.1186/s12911-017-0559-5

**Published:** 2017-12-20

**Authors:** Hong Song, Lei Chen, RuiQi Gao, Iordachescu Ilie Mihaita Bogdan, Jian Yang, Shuliang Wang, Wentian Dong, Wenxiang Quan, Weimin Dang, Xin Yu

**Affiliations:** 1 School of Software, Beijing Institute of Technology, Beijing, China; 2 School of Optics and Electronics, Beijing Institute of Technology, Beijing, China; 3 Peking University Sixth Hospital, Peking University Institute of Mental Health, Key Laboratory of Mental Health, Ministry of Health (Peking University), Beijing, China

**Keywords:** Functional near-infrared spectroscopy, Schizophrenia discrimination, Complex brain network analysis, Support vector machine

## Abstract

**Background:**

Schizophrenia is a kind of serious mental illness. Due to the lack of an objective physiological data supporting and a unified data analysis method, doctors can only rely on the subjective experience of the data to distinguish normal people and patients, which easily lead to misdiagnosis. In recent years, functional Near-Infrared Spectroscopy (fNIRS) has been widely used in clinical diagnosis, it can get the hemoglobin concentration through the variation of optical intensity.

**Methods:**

Firstly, the prefrontal brain networks were constructed based on oxy-Hb signals from 52-channel fNIRS data of schizophrenia and healthy controls. Then, Complex Brain Network Analysis (CBNA) was used to extract features from the prefrontal brain networks. Finally, a classier based on Support Vector Machine (SVM) is designed and trained to discriminate schizophrenia from healthy controls. We recruited a sample which contains 34 healthy controls and 42 schizophrenia patients to do the one-back memory task. The hemoglobin response was measured in the prefrontal cortex during the task using a 52-channel fNIRS system.

**Results:**

The experimental results indicate that the proposed method can achieve a satisfactory classification with the accuracy of 85.5%, 92.8% for schizophrenia samples and 76.5% for healthy controls. Also, our results suggested that fNIRS has the potential capacity to be an effective objective biomarker for the diagnosis of schizophrenia.

**Conclusions:**

Our results suggested that, using the appropriate classification method, fNIRS has the potential capacity to be an effective objective biomarker for the diagnosis of schizophrenia.

## Background

Schizophrenia [1] is a mental disorder characterized by abnormal social behavior and failure to understand what is real. Common symptoms include false beliefs, unclear or confused thinking, hearing voices that others do not hear, reduced social engagement and emotional expression, and a lack of motivation. It not only produce great pain to the patients but also bring a heavy burden to their family.

fNIRS is a haemodynamic-based technique for the assessment of functional activity in the human brain [2]. Based on the tight coupling of neural activity and oxygen delivery [3], changes in the concentration of oxygenated and deoxygenated haemoglobin are noninvasively measured by fNIRS and taken as indicators for cortical activation. The typical fNIRS signal observed after neural activation is a decrease of deoxygenated accompanied by an increase of oxygenated comparable in time course to the blood oxygenation level dependent signal of fMRI [4]. fNIRS provides comprehensive information about haemodynamics consisting of oxygenated, deoxygenated and changes in total haemoglobin. It is characterised by its straightforward application which resembles in the outward appearance more an electroencephalogram. Thus, the data collection is comfortable for the subjects because of the less constrictive measurement circumstances which probably lead to more ecologically valid conditions than in other neuroimaging methods [5]. These inherent advantages accompanied by the rapid developments in technology and methodology enabled fNIRS to easily enter psychological, psychiatric and basic research on children, adults and elderly subjects.

Because the symptom of schizophrenia is similar with other diseases, such as depression and anxiety. Doctors can only use the information of genetic predisposition, substance abuse, living conditions and prenatal stressors to predict the schizophrenia is triggered or not. Usually it is not generate immediately but takes years for the disease to surface. So design a computer aided identification method can help improving the doctor’s diagnostic result. With that being the case, many patients can have access to a proper medication, as such, the wellbeing of the patients and the medical quality of hospitals will increase.

During the past several years, many studies have applied the fNIRS technique to investigate the brain activation patterns in patients with schizophrenia. Converging evidence suggests schizophrenia patients are often associated with reduced activities and inappropriate activity timing around the bilateral prefrontal cortex during a verbal fluency task or other cognitive tasks [6]. Based on these findings, some studies have attempted to apply the fNIRS signal as a diagnostic tool with different pattern recognition methods. In [7], authors measured the changes of the oxy-Hb signal during multiple cognitive tasks from two fNIRS channels located in the bilateral prefrontal areas and then applied stepwise linear discriminant analysis to distinguish patients with schizophrenia from healthy subjects. They separated the total sample into two groups, and each group consisted of 60 subjects (including 30 patients with schizophrenia and 30 age-and gender-matched healthy controls). The experimental results demonstrated that there was an accuracy rate of 88.3% for classification in the first group, and the discrimination function derived from the first group correctly differentiated 75% of the subjects in the second group. To integrate spatial and temporal information in multichannel fNIRS, [8] employed a novel probabilistic pattern recognition method called Gaussian process classifier for the diagnostic classification of schizophrenia. Using the temporal patterns of fNIRS data measured during a working memory task, an overall accuracy of 76% was achieved in a group of 80 samples. And [9] applied a 52 channel fNIRS system to identify the significantly different regions in the prefrontal cortex during a verbal fluency test and then utilized a k-means clustering method for discriminant analysis between schizophrenia patients and healthy subjects. The results indicated 68.69% and 71.72% of the participants were correctly classified as schizophrenic or healthy subjects with all 52 channels and six significantly different channels, respectively. And [10] proposed a method using principal component analysis and SVM to discriminate patients with schizophrenia from health controls using a large sample of 52 schizophrenia patients and 38 healthy controls. They achieved a satisfactory classification with the accuracy of 93.33%, 100% for schizophrenia samples and 84.62% for healthy controls.

Human brain network is one of the complex networks [11, 12]. Researchers have used the complex network theory [13, 14] to construct the brain network, then analyze the constructed brain network using complex network theory and calculate index of the brain network for further study. The brain network can be divided into structural and functional brain network [15, 16]. The nodes and edges are two key elements in the brain network. Diffusion tensor imaging and diffusion spectrum imaging [17, 18] are two imaging techniques used in structural brain network. Since the two techniques can track the direction of the mental fiber electrical signal, the structural brain network is considered as directional. The definition of the node of functional brain network is changing with different imaging techniques. Generally we define the connections between nodes by calculating pearson correlation or partial correlation which is used to describe the statistical significance of functional brain signals over a period of time. The functional network is non-directional since the correlation between nodes only reflects their statistical significance, no causal relationship.

In this paper, we designed a cognitive task and recruited a group of subjects to perform this task. The group included 42 schizophrenia patients and 34 healthy controls and a 52 multichannel fNIRS system was used to examine the hemodynamic signals in the bilateral prefrontal and superior temporal cortices during the cognitive task. Then we used CBNA to extract the effective features between schizophrenia patients and healthy controls. Finally we trained the SVM classifier and evaluated it with leave-one-out cross validation. The results show that the proposed approach has the high potential to be a promising clinical tool in the objective diagnosis and treatment of psychiatric disorders.

## Methods

### DataSet description

**Fig. 1:**
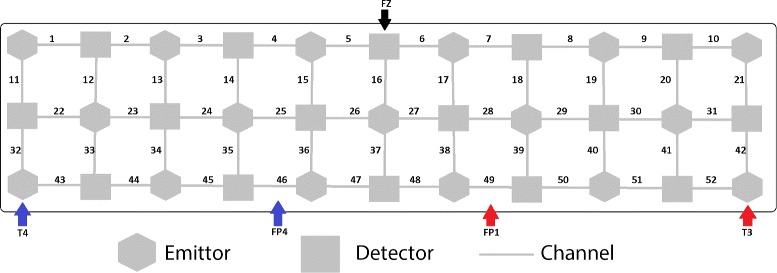
Ten to twenty system channel map for the 52 channels

The dataset was provided by Peking University Sixth Hospital. The fNIRS measurements were conducted with a 52 multichannel fNIRS System (ETG-4000, Hitachi Medical Co., Japan). In the system, 33 probes (17 emitters and 16 detectors) were stabilized on the scalp and arranged as a 3 × 11 array, which was positioned according to the international 10–20 system. The recording channels were established between each pair of emitters and detectors, which resulted in 52 channels total. Specifically, the detector between Channel 5 and 6 was located at Fz, Channel 46 and 49 were placed in Fp2 and Fp1, and the emitters which were close to Channel 43 and 52 were fitted around T4 and T3, respectively. Fz, Fp2, Fp1, T4 and T3 are the reference electrode positions in the international 10-20 system, shown in Fig. 1. Thus, the fNIRS probe set covered the entire prefrontal cortex and some regions of the superior temporal cortex. The relative changes in oxy-Hb and deoxy-Hb were measured using a reflectance mode with two different wavelengths (695 and 830 nm) of near-infrared light. The relative changes in total-Hb were equal to the sum of oxy-Hb and deoxy-Hb. The temporal resolution of fNIRS was set to 0.1 s.

The dataset included 42 patients with schizophrenia (mean age: 31 ± 12 years, female/male: 26/16) and 34 ageand sex-matched healthy controls (mean age: 33 ± 10 years, female/male: 20/14). All subjects were right-handed and native Chinese speakers. The diagnosis for schizophrenia was based on the Structured Clinical Interview for the DSM-IV (American Psychiatric Association, 1994). The healthy controls were enrolled through the local community and then assessed to confirm no history of psychiatric or neurologic disorders. This study was conducted in accordance with the Declaration of Helsinki and was approved by the ethics committee of Peking University Sixth Hospital. All subjects provided written informed consent after the experimental procedure had been fully explained.

**Fig. 2:**
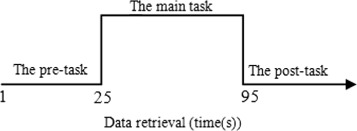
The time variance in the data retrieval

The experiment was performed in a quiet environment. All subjects were required to maintain emotionally stability prior to the experiment and to avoid moving the head as much as possible during the measurement. We designed a one-back memory task. The task comprised a 5s pre-scan and a 25s waiting period, a 70s task period, and a 50s post-task baseline period. We can see it in Fig. 2. During the pre- and post-task baseline periods, the subjects were required to stare the screen. During the task period, they were instructed to press a button with their right index finger whether or not the current image presented on the screen was the same as the previously shown one. The images were changed every 2s and the rendering time was 0.5s, there were a total of 29 figures shown. A detailed description of this experimental procedure could be found in elsewhere [19].

### Schizophrenic discrimination method

**Fig. 3:**
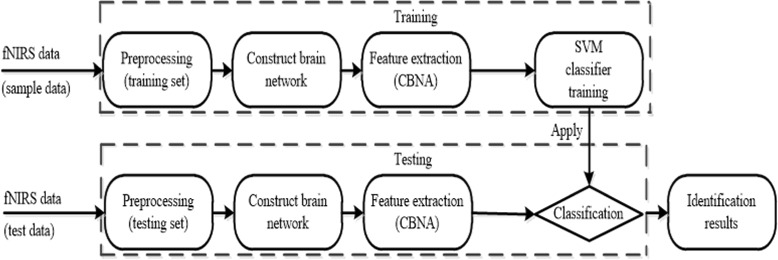
The framework of schizophrenia discrimination

The process of schizophrenia discrimination includes preprocessing the fNIRS data, constructing brain network, feature extraction, training the classifier, cross validation and testing. The flowchart of schizophrenia discrimination process is shown in Fig. 3. In this paper, CBNA is used to extract the feature eigenvalues and SVM is used as the classifier.

#### Preprocessing data

**Fig. 4:**
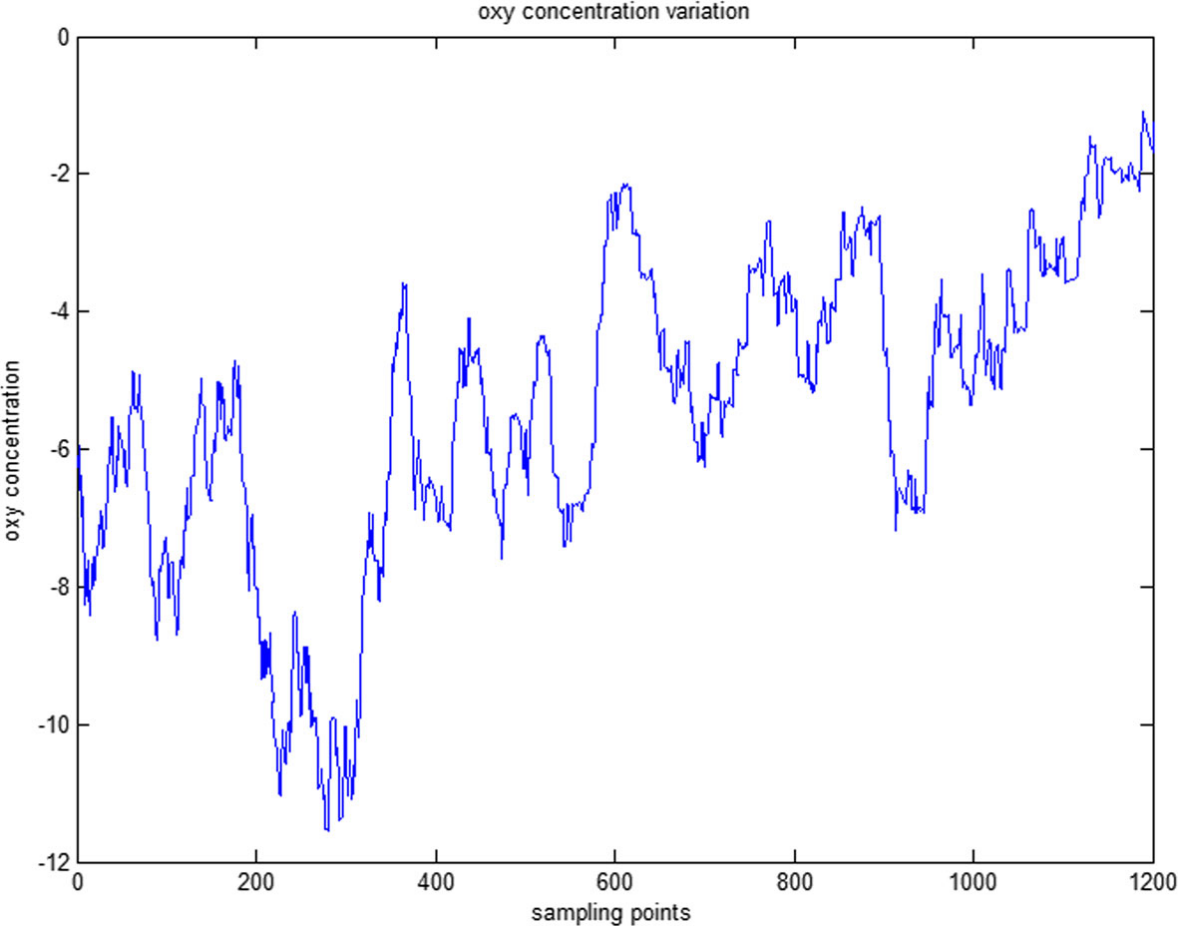
Original oxy hemoglobin concentration

**Fig. 5:**
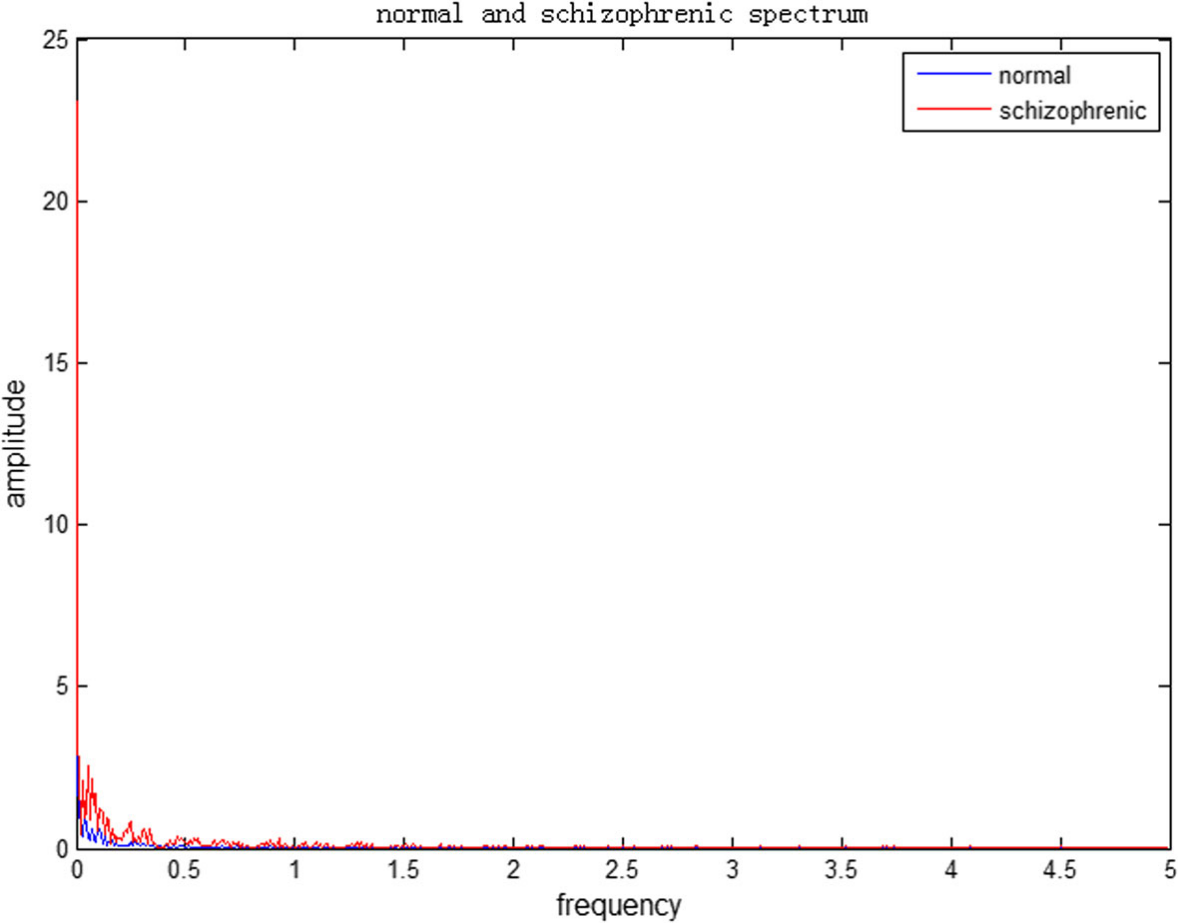
The fourier transform of original oxy hemoglobin concentration of normal and schizophrenia people

**Fig. 6:**
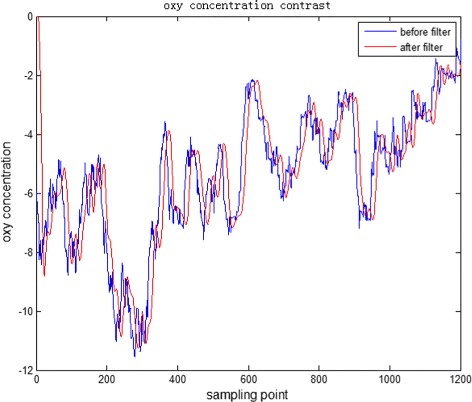
Oxy hemoglobin concentration before and after low-pass filtering

The original fNIRS data of a schizophrenia patient is shown in Fig. 4, the blue line plots the oxy hemoglobin concentration variation of healthy person. In order to reduce the effects of the high frequency noise, low-pass filtering is used to do data preprocessing. Before low-pass filtering, We first use Fast Fourier transform to do spectrum analysis to find which frequency the fNIRS exists in. Figure 5 shows the Fast Fourier transform result of original oxy hemoglobin concentration variation of healthy person and schizophrenia patient, blue line represents the healthy person and red line represents the schizophrenia patient. It is obviously that the frequency of fNIRS signal mainly exists in 0.0–0.5 HZ, so a low-pass filter with the cut-off frequency 0.5 Hz is designed to do the filtering. The result of the low-pass filtering is shown in Fig. 6. The blue line represents original data and red line represents processed data. It can be seen that the curve after low-pass filter is smoother than before, some high frequency noise is reduced. This reduced the effects of high frequency noise and improved the classification accuracy.

#### Construct the brain network

**Fig. 7:**
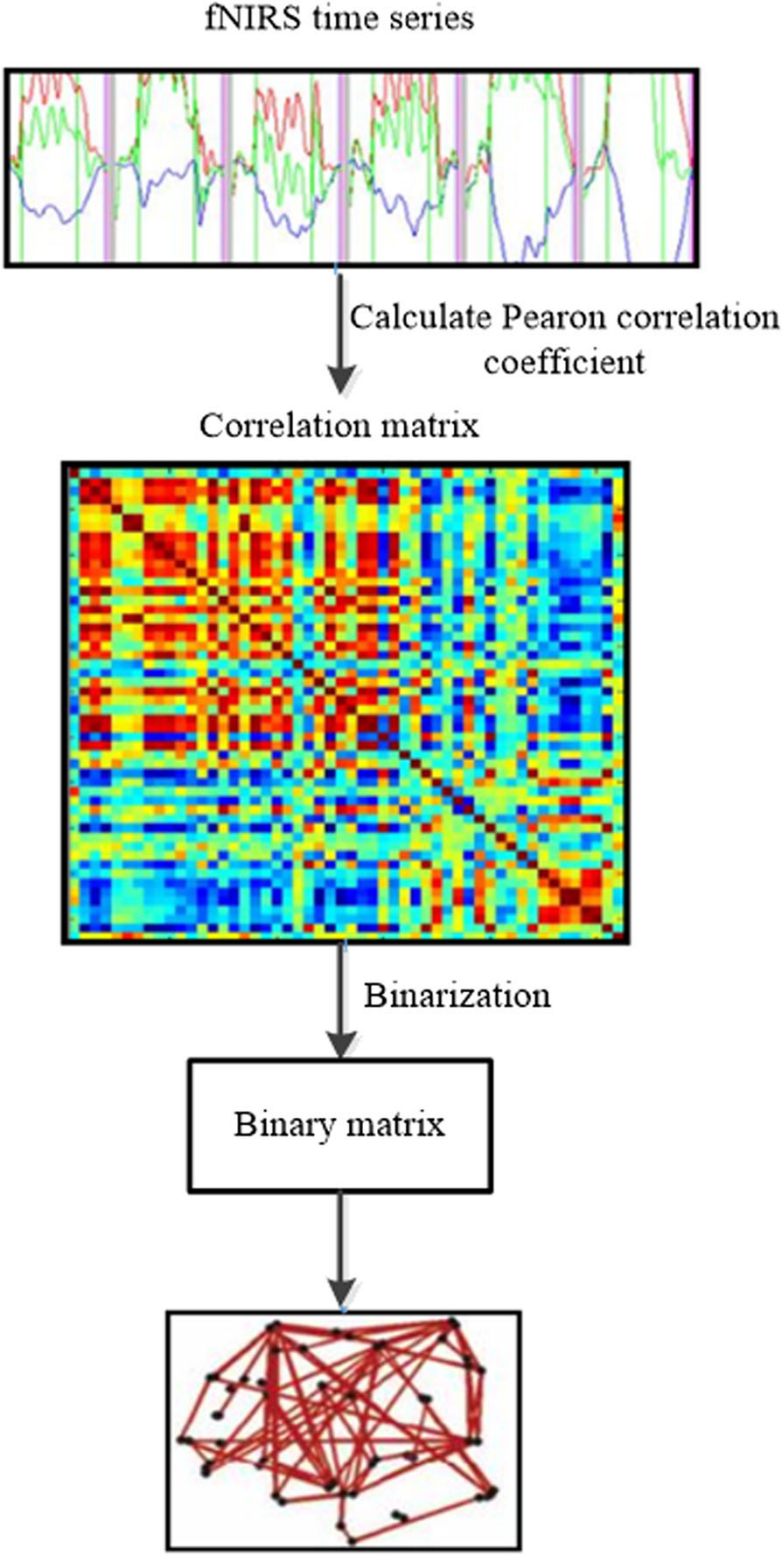
The flow chart of functional brain network construction

The process of a functional brain network construction is shown in Fig. 7. During the construction process, defining the edge and node of the brain network are two important steps. Nodes in the brain network correspond to the measured 52 channels of fNIRS. The Pearson correlation coefficient of fNIRS time series between different nodes is usually calculated to quantify the relationship between them. The edge is defined by setting an appropriate threshold to binarize the connection relationship of the nodes.

The Pearson correlation coefficient is calculated as follows, take the *i* ^*th*^ and *j* ^*th*^ channel’s fNRIS data as variable *X*,*Y*(*i*,*j*=1,2…52,*i*≠*j*), the Pearson correlation coefficient is *r*_*i*,*j*_, then

1 $$\begin{array}{@{}rcl@{}} r_{i,j} = \frac{cov(X,Y)}{\sigma_{X}\sigma_{Y}} &=&\frac{E(X-\mu_{X})(Y-\mu_{Y})}{\sigma_{X}\sigma_{Y}} \\ &=& \frac{E(XY)-E(X)E(Y)}{\sqrt{E(X^{2})-E^{2}(X)E(Y^{2})-E^{2}(Y)}} \end{array} $$

Where *c* *o* *v*(*X*,*Y*) is the covariance, *σ*_*X*_, *σ*_*Y*_ are the standard deviation, *μ*_*X*_, *μ*_*Y*_ are the mean of variable *X* and *Y* respectively. The Pearson correlation coefficient of two variables *X* and *Y* is equal to the covariance divided by the product of the standard deviation of the two variables.

**Fig. 8:**
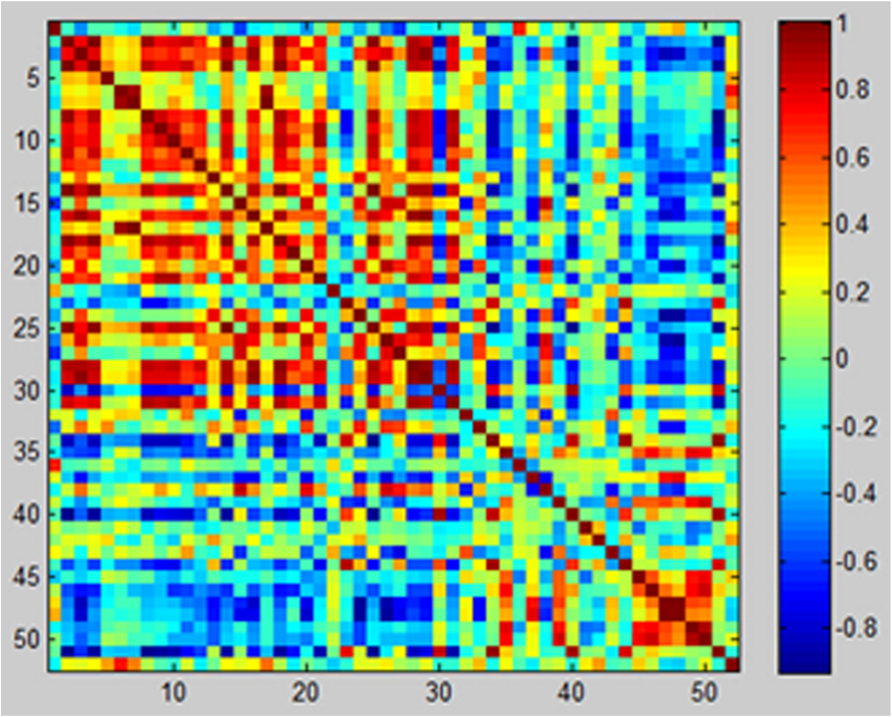
Pearson correlation coefficient matrix of a schizophrenic patient after color rendering

From the calculation process of Pearson correlation coefficient, we can get a Pearson correlation coefficient matrix with a row and column which have 52 dimensions for a subject. Each element in the matrix is a Pearson correlation coefficient value. When *i*=*j*, the Pearson correlation coefficient is 1. Here we calculated the Pearson correlation coefficient of 76 subjects and constructed 76 correlation coefficient matrices. Fig. 8 shows the result of Pearson correlation coefficient matrix of a schizophrenic patient after color rendering.

After quantizing the relationship between 52 channels,we got the Pearson correlation coefficient matrix, it is necessary to choose an appropriate threshold *T* in order to construct the edge of the nodes. Whether there is an edge connection between two nodes is depend on the Pearson correlation between two channels.If the absolute value of the Pearson correlation coefficient is greater than the threshold, there is an edge between the corresponding nodes, and vice versa.

2 $$ e_{i,j}= \left\{ \begin{array}{ll} 1,&|r_{i,j}|\ge T\\ 0,&|r_{i,j}|<T \end{array} \right.  $$

We use sparsity as a measurement of the threshold *T*. Sparsity is the ratio of the actual number of edge to the possible maximum number of edge in matrix. There is no quantitative relationship between sparsity *S* and threshold *T*. If the sparsity of the binarized matrix is set to 50%, when binarizing the Pearson correlation matrix, *T* is the median of the ascending correlation coefficient. When the correlation coefficient is greater than the median, it has edge and vice verse. The actual number of edge is just half of the maximum possible one. In the study of the brain network, most researchers use sparsity with fixed interval to study the topological properties of the brain network with multiple thresholds. Here we set the sparsity range from 1 to 50% to ensure the sparseness of the network.

After binarization, we get the binary matrix which is corresponding to the brain network matrix. Thus we get the brain network of 76 subjects.

#### Feature extraction based on CBNA

**Fig. 9:**
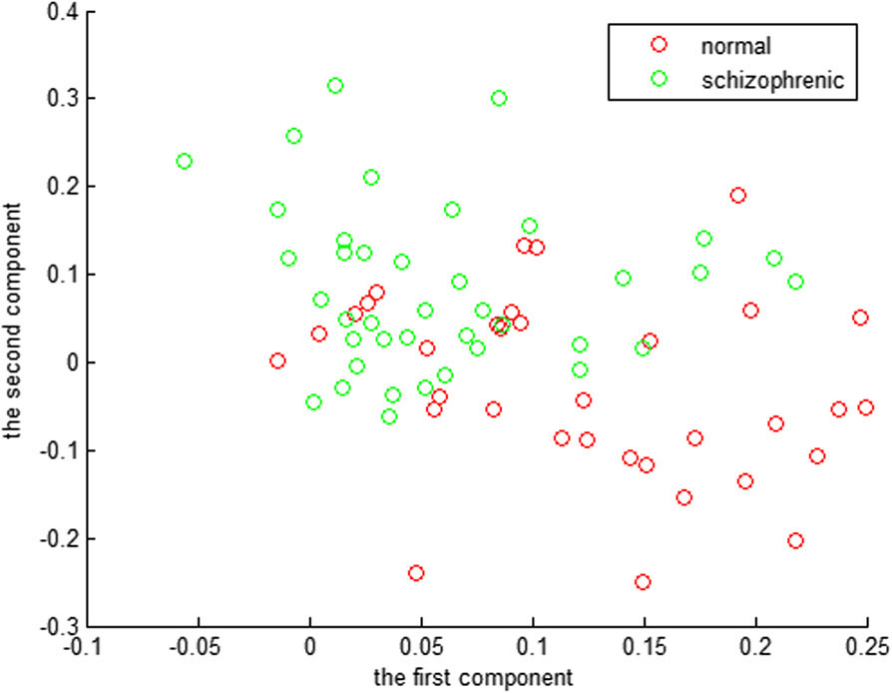
Result of eigenvectors constructed with the attribute of node degree when the threshold is set to 0.21

After get the brain network of 76 subjects, the basic attribute index of the network is calculated by using the formula of basic attribute value in complex network theory. The basic attributes we used here are the degree of node, clustering coefficient, local efficiency and global efficiency. In this way we get a 52-dimensional eigenvector of the above four attributes. Since the threshold is changed from 0.01 to 0.5 with an interval of 0.01, it is necessary to select eigenvector with significant differences in each attribute from the 50 thresholds to make subsequent classification decisions. After reduce the dimension of eigenvector of each attribute, we find there is a significant difference of the node degree when the threshold is set to 0.21. Thus, we choose the attribute of the node degree at threshold 0.21 to construct the final eigenvector. Fig. 9 shows the result of eigenvectors constructed with the attribute of node degree when the threshold is 0.21. It can be seen that the eigenvector between the healthy control and schizophrenic has significance difference.

#### SVM-based classifier

The SVM is a learning machine for a two-class classification problem [20]. It is proposed by Vapnik as an extension of statistical learning theory. Due to its ability to handle high-dimensional data and could acquire high accuracy in the classification, SVM has been widely used in many areas.

SVM conceptually implements the idea that vectors are non-linearly mapped to a high dimension feature space. In the feature space, a linear separation surface is created to separate the training data by minimizing the margin between the vectors of the two classes. The training ends with the definition of a decision surface that divides the space into two sub-spaces. Each sub-space corresponds to one class of the training data. Once the training is completed, the test data are mapped to the feature space. A class is then assigned to the test data depending on which sub-space they are mapped to. In this paper, a SVM toolkit named libsvm written by Lin Chih-Jen from Taiwan University [21] is used. A radial basis function is selected as a kernel function and parameters are kept as default values.

Cross validation is frequently used in classification problems, which mainly divided into *K* fold cross validation and the leave-one-out cross validation. Here we use the leave-one-out cross validation to evaluate the classification result. Suppose there are *N* samples, leave-one-out cross validation continues *N* rounds, each round one sample will be as a test sample, the rest *N*−1 samples are as the training sample. After *N* rounds of cross validation, each round will get a classification accuracy. Finally, the average classification accuracy will be acquired for *N* rounds. leave-one-out cross validation can effectively reduce the classification accuracy error caused by the inefficient sample. Here the dataset we used include 34 healthy persons and 42 schizophrenic patients. In the leave-one-out cross validation, the dataset is separated into 76 samples, every sample will be as a test sample and the rest 75 samples will be a training set, continues 76 rounds.

## Results and discussion

The testing result of schizophrenics and healthy controls is shown in Table 1, where 39 of the 42 schizophrenia and 26 of 34 health controls were discriminated successfully on Oxy-Hb/Deoxy-Hb signal. The method based on CBNA and SVM successfully discriminated 65 (39 schizophrenia and 26 healthy persons) signals with an overall accuracy of 85.5% for fNIRS classification on testing set. And on total signal, where 39 of the 42 schizophrenia and 22 of 34 health controls were discriminated successfully. The method successfully discriminated 61 (39 schizophrenia and 22 healthy persons) signals with an overall accuracy of 80.3%.

**Table 1:** The finally testing result

	Classification accuracy	Specificity	Sensitivity
Oxy-Hb	85.5%	76.5%	92.8%
Deoxy-Hb	85.5%	76.5%	92.8%
Total	80.3%	64.7%	92.8%

Our study was a binary classification, and we first defined the class of schizophrenia patient as positive and the class of healthy control as negative. Then, TP is the number of schizophrenia patients correctly predicted; TN is the number of healthy controls correctly predicted; FP is the number of healthy controls classified as schizophrenia patients; and the FN is the number of schizophrenia patients classified as healthy controls. Finally, the performance of a classifier can be quantified by using the ACC, SS and specificity or TNR; These measures are defined as follows:

3 $$\begin{array}{@{}rcl@{}} ACC&=&\frac{TP+TN}{TP+FP+TN+FN} \end{array} $$

4 $$\begin{array}{@{}rcl@{}} SS&=&\frac{TP}{TP+FN} \end{array} $$

5 $$\begin{array}{@{}rcl@{}} TNR&=&\frac{TN}{TN+FP} \end{array} $$

ACC represents the ration between correctly classified samples and total samples. SS represents the ratio between correctly classified schizophrenic patients and total schizophrenic patients. TNR represents the ratio between correctly classified health controls and total health controls. Therefore, a good fNIRS-aided diagnostic classifier is assumed to have larger ACC and TNR values.

After 76 rounds of cross validation, there are totally 11 misclassified cases on Oxy-Hb signal. Including 8 cases of normal people and 3 cases of schizophrenic patients. The classification accuracy is 85.5%, specificity is 76.5%, sensitivity is 92.8%. Table 2 shows more details about the testing results on Oxy-Hb signal. For the Deoxy-Hb signal, there are also 11 misclassified cases. Including 8 cases of normal people and 3 cases of schizophrenic patients. The classification accuracy is 85.5%, specificity is 76.5%, sensitivity is 92.8%, the same as Oxy-Hb signal. For the total signal is shown in Table 3. There are 15 misclassified cases. Including 12 cases of normal people and 3 cases of schizophrenic patients. The classification accuracy is 80.3%, specificity is 64.7% and sensitivity is 92.8%. Here we choose Oxy-Hb signal to discriminate schizophrenic. This accuracy is especially satisfactory for the discrimination.

**Table 2:** Testing result of schizophrenic and healthy on Oxy-Hb signal

CBNA+SVM			Classified results	
			1(Schizophrenia)	-1(healthy)
			47	29
Data set	1(Schizophrenia)	42	39	3
	-1(healthy)	34	8	26
Accuracy of schizophrenia (SS)			39/42=92.8%	
Accuracy of healthy((TNR))			26/34=76.5%	
Classification accuracy(ACC)			65/76=85.5%

**Table 3:** Testing result of schizophrenic and healthy on total signal

CBNA+SVM			Classified results	
			1(Schizophrenia)	-1(healthy)
			61	25
Data set	1(Schizophrenia)	42	39	3
	-1(healthy)	34	12	22
Accuracy of schizophrenia (SS)			39/42=92.8%	
Accuracy of healthy((TNR))			22/34=64.7%	
Classification accuracy(ACC)			61/76=80.3%	

## Conclusion

Our study demonstrated that the designed task is an effective experimental paradigm. Compared with healthy controls, the multichannel fNIRS results on the sample confirmed that schizophrenia patients in the Chinese population had significant lower brain activation over the prefrontal cortex and superior temporal cortex. Finnally, we achieved a considerable overall classification accuracy of 85.5% (65/76) using the SVM classifier and CBNA based feature selection on the oxy-Hb signal. Thus, SVM had the good classification performance especially after performing the CBNA based feature selection. Our results illustrated that, by using the appropriate classification method, fNIRS represents a promising diagnostic tool to differentiate schizophrenia patients from healthy controls.

## Abbreviations

ACC: Accuracy
CBNA: Complex brain network analysis
Deoxy-Hb: Deoxygenated hemoglobin
FN: False negative
fNIRS: Functional near-infrared spectroscopy
FP: False positive
LN: Letter-number
OSH: Optimal separating hyperplane
Oxy-Hb: Oxygenated hemoglobin
SVM: Support vector machine
TP: True positive
TN: True negative
TNR: True negative rate
VFT: Verbal fluency test
